# A New Function for Perivascular Adipose Tissue (PVAT): Assistance of Arterial Stress Relaxation

**DOI:** 10.1038/s41598-020-58368-x

**Published:** 2020-02-04

**Authors:** Stephanie W. Watts, Emma D. Flood, Hannah Garver, Gregory D. Fink, Sara Roccabianca

**Affiliations:** 1 Department of Pharmacology and Toxicology, Michigan State University, East Lansing, MI 48824-1317 USA; 2 Department of Mechanical Engineering, Michigan State University, East Lansing, MI 48824-1317 USA

**Keywords:** Physiology, Cardiovascular biology, Vasodilation

## Abstract

In health, PVAT secretes anti-contractile factors that relax the underlying artery. PVAT’s contributions to vascular function include more than production of vasoactive substances. We hypothesized that PVAT benefits the artery by assisting the function of stress (–induced) relaxation. Thoracic aorta rings from Sprague Dawley rats were mounted in isolated tissue baths with (+) and without (−) PVAT. A cumulative length tension (0–6 grams) was generated. The tension to which the tissue stress relaxed over 30 minutes was recorded; the tension lost was stress relaxation. The presence of PVAT increased the amount of stress relaxation (final tension in mgs; aortic ring −PVAT = 4578 ± 190; aortic ring + PVAT = 2730 ± 274, p < 0.05). PVAT left attached but not encompassing the aorta provided no benefit in cumulative stress relaxation (aortic ring +/− PVAT = 4122 ± 176; p > 0.05 vs −PVAT). A PVAT ring separated from the aorta demonstrated more profound stress relaxation than did the aortic ring itself. Finally, PVAT-assisted stress relaxation was observed in an artery with white fat (superior mesenteric artery) and in aorta from both male and female of another rat strain, the Dahl S rat. Knowledge of this new PVAT function supports PVAT as an essential player in vascular health.

## Introduction

For decades, perivascular adipose tissue [PVAT^1^] was routinely dissected from the blood vessel when studying contractility in isolated vessels. Caroline Pond^2^ states why: “human and comparative anatomists still regarded it as too inconsistent and inconsequential to be worthy of topographic, functional or evolutionary study. It was always dissected off vessels…”. This changed with the study by Soltis and Cassis, published in 1991^3^, that supported that the presence of PVAT changed vessel reactivity to norepinephrine (NE).

In 2002, Lohn *et al*. extended this finding in a simple, provocative and compelling way. Transfer of physiological buffer, in which PVAT from a healthy rat was incubated, to isolated, contracted rat thoracic aorta with no PVAT caused a direct relaxation^4^. They concluded there must be a transferable substance made within the PVAT that caused relaxation. This finding reaffirmed the idea that PVAT was anti-contractile. Since this study, investigators have reproduced that the presence of PVAT in healthy tissue is ‘anti-contractile’^5–11^. Gollasch uses the term “adipose-vascular coupling” for the interaction of PVAT with the vessel^12^ and, in health, this coupling is beneficial. PVAT is best known for the vasorelaxant substances it makes and secretes. These substances are as diverse as a gas (hydrogen sulfide, nitric oxide) to a protein (adiponectin)^13–19^. It is important to note PVAT also makes and secretes contractile substances including angiotensin II and superoxide^9,20–22^. The loss of the ‘anti-contractile’ nature of PVAT, including promotion of endothelial dysfunction, has been observed in vasculature from multiple models of cardiovascular disease – diabetes, hypertension – and in multiple species that include the human^6,7,23–25^. PVAT has been argued to be both an instigator and a protective compensator in disease^26–30^ at the levels of vascular remodeling^31–33^ and arterial stiffness^22,34^.

While important, the totality of these studies consider PVAT primarily as a tissue that secretes vasoactive substances. Here, we highlight an unappreciated function of PVAT, namely to support stress-induced relaxation. Stress relaxation, a loss of tone after a stretch is applied, is a beneficial event by reducing vessel tension*. In vivo* tension is proportional to luminal pressure **x** radius, mechanisms that reduce tension over time with a fixed radius represent a homeostatic move to keep stress constant. We used the thoracic aorta of the rat as our primary model because the PVAT around this artery is discrete. The PVAT in this artery is primarily brown fat^35,36^. We test the hypothesis that PVAT provides structural benefit to the artery by assisting the function of stress relaxation. This was done by using rings of artery with PVAT intact and with neighboring arterial rings with PVAT removed. By comparing the two, we could determine if stress relaxation was PVAT-dependent. While the focus was primarily on the thoracic aorta of the Sprague Dawley rat, we also tested this hypothesis in an artery with a primarily white fat PVAT, the superior mesenteric artery^37^, to determine if stress relaxation was fat-type dependent. Finally, we determined whether PVAT-dependent stress relaxation could be more generalized to the rat and female by using the thoracic aorta from the Dahl S male and female rat^38^. The present studies support the ability of PVAT to assist stress relaxation.

## Results

### PVAT assists stretch-induced relaxation in the isolated thoracic aorta

A length-tension curve from the passive tensions of 0 to 6 grams was generated to investigate how tissue responds to increasing stretch in the presence and absence of PVAT. This range was chosen as it encompasses the passive tension (2–6 grams) that result in a maximum active contraction to phenylephrine (PE). Tissues were placed under increasing, cumulative magnitudes of force (stretch, in grams), and allowed to relax for 30 minutes; this was stress relaxation. At the end of this 30-minute period, the final tension was recorded and tissues were challenged with the α_1_ adrenergic agonist PE to determine viability. The whole thoracic aorta was used, with sections from close to the arch and diaphragm randomized such that they were represented equivalently in each experimental group. Tissues are referred to as: aortic ring +PVAT (abbreviated +PVAT; rings with PVAT fully attached), aortic ring −PVAT (−PVAT; rings with PVAT removed), PVAT ring (just the ring of PVAT) or aortic ring +PVAT ring (PVAT ring removed and added back) in the experiments described below. These different experimental groups were generated from the same animals such that a direct comparison of responses could be made.

**Figure 1:**
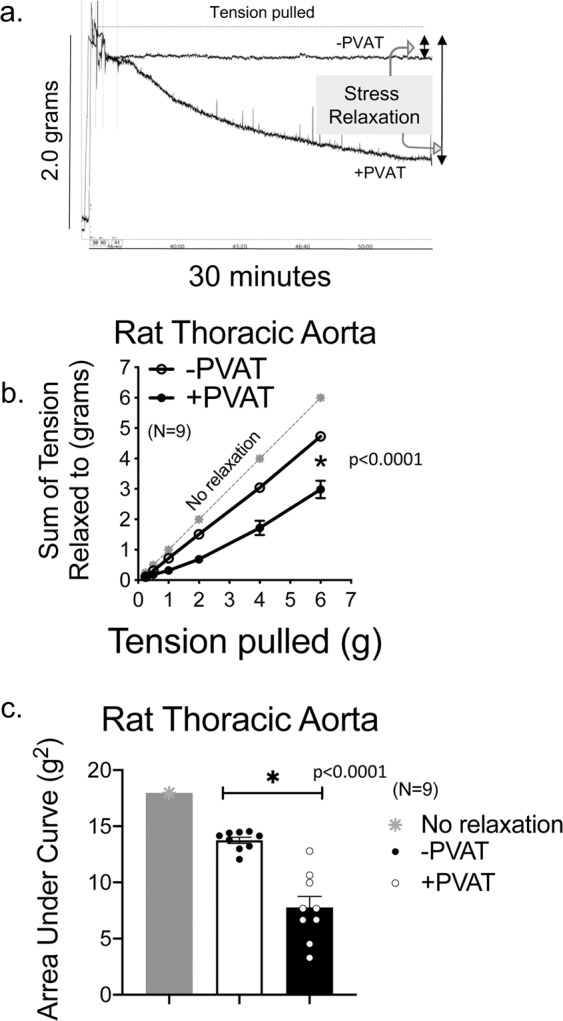
PVAT assists stress relaxation in thoracic aorta. **(a**) A representative tracing of isometric tension of aortic ring + and −PVAT over a 30-minute stretch step in a protocol using PE challenges between each stretch step. (**b**) Quantitation of the cumulative tension achieved with each stretch step vs no relaxation (gray line). (**c**) Quantitation of the area under the curve for aortic rings +PVAT and aortic rings −PVAT *vs* that of no relaxation (gray). Final mass: aortic rings +PVAT = 61.7 +/- 3.7 mgs; aortic rings −PVAT = 7.9 +/- 0.5 mg. Points/bars represent means +/- SEM for the number of animals in parentheses. *Significant differences as determined by a two-tail unpaired Students t-test (p value reported in figure) for maximum values.

The presence of PVAT around an aortic ring from a male Sprague Dawley rat markedly increased the amount of stress relaxation, illustrated by the representative tracing in Fig. 1a. The sum of the tensions achieved with increasing passive stretch is quantified in Fig. 1b. The grey line (a bisector) represents what the results would be if no stress-induced relaxation occurred. The aortic ring achieved a lower sum of resting tension when PVAT was present than absent (+PVAT vs −PVAT in graph), though aortic rings −PVAT demonstrated clear stress relaxation in response to stretch (compare −PVAT to gray line). The area under each of these three curves (no relaxation, −PVAT and +PVAT) was quantified for each tissue as another measure of the effect of PVAT and these results are shown in Fig. 1c. The presence of PVAT encompassing the vessel reduced this value, another way of demonstrating that PVAT promoted overall arterial stress relaxation.

**Figure 2:**
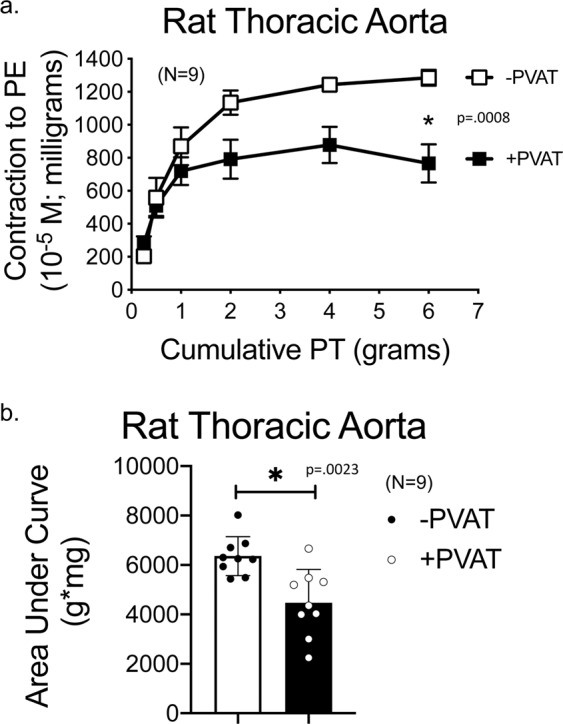
PVAT anti-contractile effect is present through a range of passive tensions. (**a**) Quantitation of the magnitude of PE-induced contraction with each stretch step. (**b**) Quantitation of the area under the curve for aortic rings +PVAT and aortic rings −PVAT. Tissue masses are stated in legend for Fig. 1. Points/bars represent means± SEM for the number of animals in parentheses. *Significant differences as determined by a two-tail unpaired Students t-test (p value reported in figure) for maximum values.

Figure 2 depicts the active response (contraction) of these same tissues to a maximum concentration of PE at each individual passive tension pulled. As observed in many studies, the presence of PVAT on the aortic ring (+PVAT) reduced the magnitude of contraction to PE. This was validated by both as absolute magnitude of maxiumum contraction (Fig. 2a) and as an area under the curve (Fig. 2b).

### Removing PVAT from length of aorta abolishes its assistance in stress relaxation

**Figure 3:**
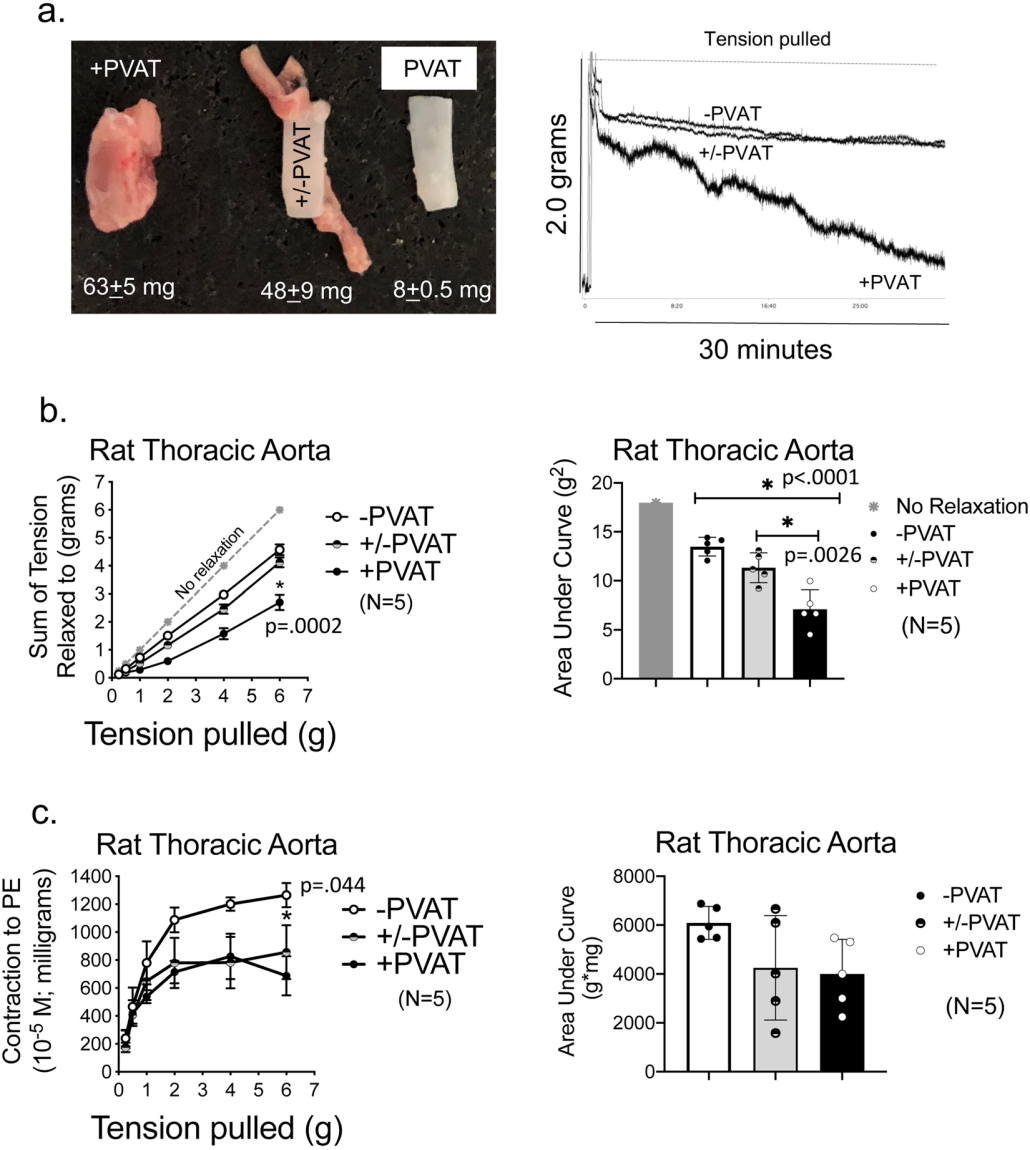
Detachment of PVAT from absolute length of ring abolishes its assistance of arterial stretch relaxation. (**a**) Image of aortic rings from the same animal +PVAT, +/−PVAT (intact only on ends), and −PVAT. Numbers beneath each vessel image is the vessel mass presented as means ± SEM for the N = 5 vessels experimented on to generate data in this figure. Quantitation of cumulative stress relaxation (**b**) and active contraction to PE (**c**) reporting absolute values (left) and area under the curve (right). Points/bars represent means ± SEM for the number of animals in parentheses. *Significant differences (p value reported in figure) as determined by a one-way ANOVA with Tukey’s post hoc determinations comparing averages as indicated by the tied bar.

We next determined if removal of PVAT from encompassing the aortic ring would alter the ability of PVAT to assist stress relaxation. The PVAT was dissected off all of the aortic ring but for at the ends, remaining in the bath during the experiment, and this sample was named aortic ring +/−PVAT (Fig. 3a). In other words, the whole PVAT burden was still present, just not all directly attached to the aortic ring. Figure 3a lists the mass of the three vessel groups in this experiment underneath a representative image of each group (aortic ring +PVAT, +/−PVAT, −PVAT). The burden of PVAT in the aortic ring +/−PVAT was statistically similar to that of +PVAT tissues (48 ± 9 vs 63 ± 5, p > 0.05 by unpaired *t* test), both groups having more mass and variability than the aortic ring −PVAT (8 ± 0.5 mg). Leaving PVAT in the bath while removed from most of the arterial surface (aortic ring +/−PVAT) *did not* provide the same assistance to stress relaxation, depicted in representative tracings in Fig. 3a right and quantified in Fig. 3b both as a line graph (left) and area under the curve (right). By contrast, the maximum response to PE in the aortic ring +/−PVAT was still reduced compared to the aortic ring −PVAT (Fig. 3C). The area under the curve for PE contraction, though, was not different between groups because of the significant variability in the response of aortic ring +PVAT (Fig. 3c).

### PVAT ring alone has profound ability to stress relax with applied stretch

**Figure 4:**
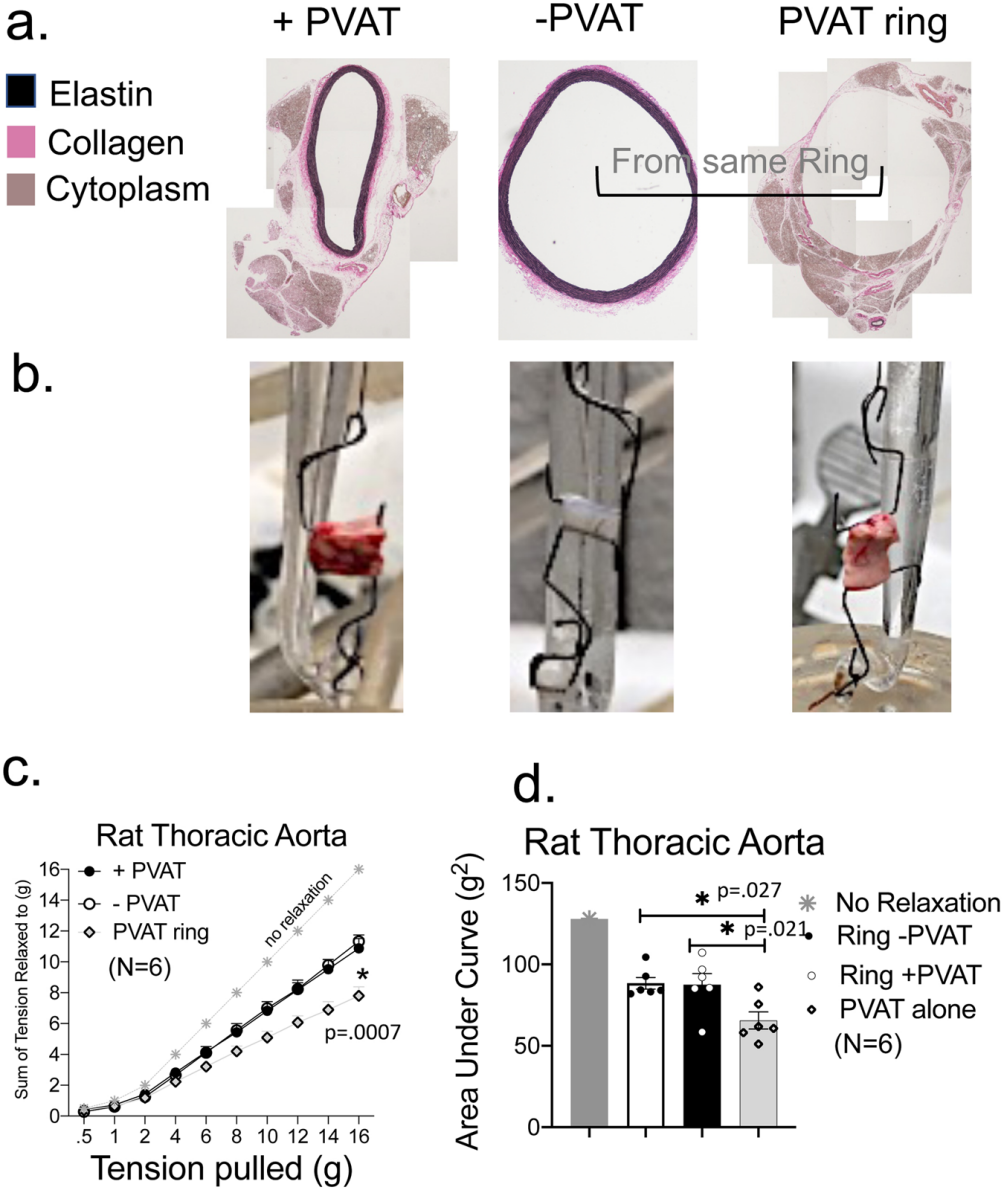
PVAT independent of vessel demonstrates profound stress relaxation. **(a**) Histology of PVAT separated from proper vessel to validate the adventitia is still present in the vessel. Representative of six separate rats. (**b**) Image of an aortic ring +PVAT, aortic ring −PVAT and PVAT ring alone when placed between two L-shaped hooks and with no tension applied. (**c**) Quantitation of the cumulative tension achieved with each stretch step in aortic ring +/− its PVAT or PVAT ring alone. (**d**) Quantitation of the area under the curve for aortic ring +PVAT, aortic ring −PVAT and PVAT ring. Final mass: aortic rings +PVAT = 51.6 ± 3.6 mg; aortic rings −PVAT = 8.1 ± 0.7 mg; PVAT ring = 38.3 ± 10.7 mg. Points/bars represent means ± SEM for the number of animals in parentheses. *Significant differences (p value reported in figure) as determined by a one-way ANOVA with Tukey’s post hoc determinations comparing averages as indicated by the tied bar.

We separated the ring of PVAT from around the aortic ring through small, discrete cuts using vannas scissors. Figure 4a shares the histology of two thoracic aortic rings from the same rat, one whole (aortic ring +PVAT) and one in which the PVAT was removed from the vessel (aortic ring – PVAT). Figure 4a (middle panels) shows the adventitial layer is still intact in the aortic ring −PVAT. The aortic ring +PVAT, aortic ring -PVAT and PVAT ring (as depicted in representative images in Fig. 4b) were mounted in isolated tissue baths and taken through the length tension curve described above but without PE challenge (we assume there is little smooth muscle in PVAT alone sample). In this experiment, we applied a greater magnitude of passive tension (to 16 grams) with the intent of finding the strength of the tissue (tension at which tissue breaks). In this protocol, aortic ring +PVAT only modestly and non-significantly reduced stress relaxation from the aortic ring −PVAT response. Both curves were lower than the line of no stress relaxation, indicating stress relaxation occurred in both groups. We expected the PVAT ring would break, an indication of poor mechanical strength. To our surprise it did not and, in fact, stress relaxed to a greater extent than the aortic ring -PVAT (Fig. 4c). This was validated in measures of the area under the curve, where the three groups – aortic ring +PVAT, aortic ring −PVAT and PVAT ring- were compared to one another (Fig. 4d).

### Adding back PVAT to the aortic ring restores PVAT-assisted stress relaxation

**Figure 5:**
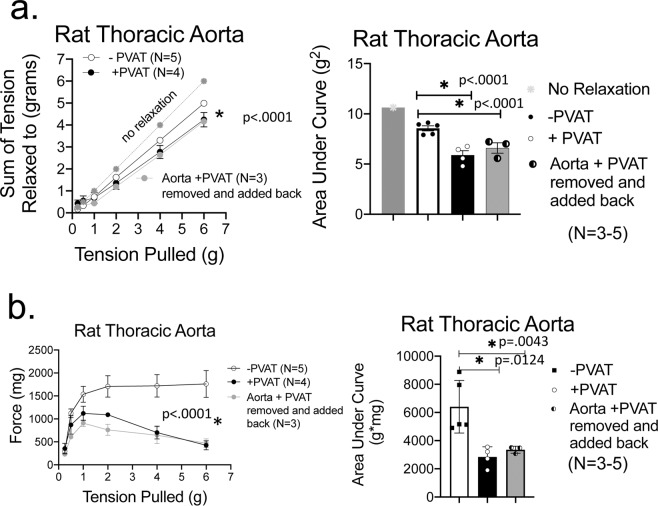
PVAT assistance of stress relaxation is independent of direct connections to artery. (**a**) Quantitation of the cumulative tension achieved with each stretch step in aortic ring +PVAT, aortic ring −PVAT and a ring in which PVAT was physically detached from artery, removed, and then placed back on artery (aorta ring +PVAT ring removed and added back) as a line graph (left) and area under the curve (right). (**b**) Response of vessels to a maximum concentration of PE after each stretch step as a line graph (left) and area under the curve (right). Final masses: aortic ring +PVAT = 35.6 ± 7.1, aortic ring −PVAT = 4.8 ± 0.9, aortic ring +PVAT ring removed and added back = 28.2 ± 2.2 mg. *Significant differences (p value reported in figure) as determined by a one-way ANOVA with Tukey’s post hoc determinations comparing averages as indicated by the tied bar.

We next examined whether placing the PVAT ring, dissected from the aortic ring, *back* around the aortic ring from which it was removed would assist stress relaxation of the aorta. In Fig. 5a, aortic rings that had PVAT directly connected(+PVAT) or the PVAT ring placed back around the aortic ring from which it was removed (aortic ring +PVAT removed and added back) showed similar stress relaxation that was greater than that in the aortic ring −PVAT, though variability was apparent. Similarly, the anticontractile nature of PVAT remained intact as long as it was in the bath **(**Fig. 5b**)**.

### PVAT from a different vessel, rat strain and sex assists arterial stress relaxation

**Figure 6:**
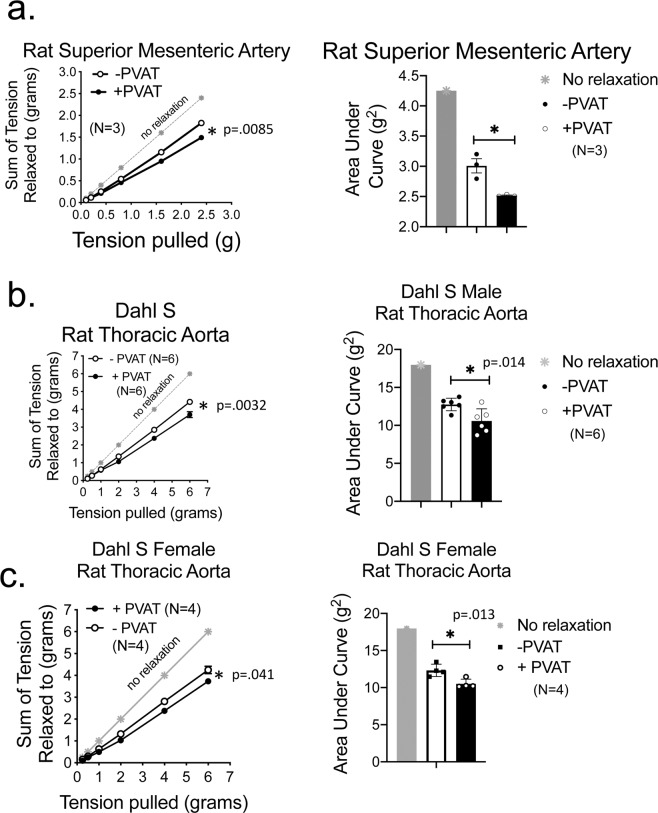
PVAT assisted stress relaxation occurs in a different artery and different rat strain. Quantitation (line graph, left; area under the curve right) of cumulative stress relaxation in the isolated superior mesenteric artery (**a**) and thoracic aorta of the Dahl S male rat (**b**) and female rat **(c**). +PVAT indicates PVAT was present, −PVAT indicates PVAT was removed. Final masses: Superior mesenteric artery +PVAT = 162 ± 17 mg; mesenteric artery −PVAT: 2.1 ± 0.45 mg; Dahl S male aortic ring +PVAT = 68 ± 4.5 mgs Dahl S male aortic ring −PVAT = 12.0 ± 1.1 mgs; Dahl S female aortic ring +PVAT = 50.7 ± 4.8 mg, Dahl S female aortic ring −PVAT = 12.3 ± 2.1 mg. Points/bars represent means ± SEM for the number of animals in parentheses. *Significant differences as determined by a two-tail unpaired Students t-test (p value reported in figure).

The same experiment as depicted in Fig. 1 was carried out in the Sprague Dawley rat superior mesenteric artery (Fig. 6a) and in the thoracic aorta of the male (Fig. 6b) and female (Fig. 6c) Dahl S rat. The PVAT around the superior mesenteric artery is primarily white fat. The presence of this PVAT enhanced/assisted arterial stress relaxation (compare +PVAT and −PVAT curves, Fig. 6a, left and right). Similarly, the PVAT of the thoracic aorta from the male and female Dahl S rat assisted stress relaxation (Fig. 6b,c, respectively). Collectively, these results support that the ability of PVAT to assist in arterial stress relaxation is neither specific to one vessel bed nor one rat strain.

## Discussion

These experiments were done with the purpose of determining whether PVAT has the ability to aid arterial stress relaxation. Using classic isolated tissue bath techniques, we discovered that not only does PVAT assist arterial stress relaxation but it itself has the profound ability to stress relax. Moreover, its presence assists arterial stress relaxation by mechanisms that do not depend on direct physical connections of PVAT to the adventitial layer below it. We do not dispute that the artery alone can stress relax upon a tension challenge, but rather add the knowledge that PVAT itself possesses the ability to stress relax.

Arteries that were encompassed by either brown (thoracic aorta) or white (superior mesenteric artery) fat both demonstrated enhanced stress relaxation in the presence of PVAT *vs* arteries with no PVAT. These findings support the idea that stress relaxation is not the property of one particular fat type or type of vessel. We also investigated two strains of rats (Sprague Dawley and Dahl S) and both male and types of vessels tested demonstrated PVAT-dependent relaxation. It will be important to determine if veins also benefit from PVAT in stress relaxation. Similarly, the coronary arteries are uniquely embedded in PVAT^39^ and their mechanical relationship with their respective PVAT will be important to understand, especially given the physical constraints on these vessels.

The ability of PVAT to assist stress relaxation of the isolated artery does not appear to depend on direct physical connections with the artery, at least for the thoracic aorta. Direct structural connections between the PVAT and adventitia have not been described. We discovered that whatever connections exist must be sparse because we could readily hold the PVAT layer apart from the adventitia and separate it from the vessel with minimal dissection. The lack of necessity of the direct physical connection of PVAT to the aorta to support stress relaxation is supported by two additional experimental outcomes. First, the PVAT ring on its own demonstrated significant stress relaxation, and is a preparation that has no smooth muscle (save for that in small vessels of the PVAT) that would be aligned in such a way that it could contribute to overall stress relaxation. Second, adding back the PVAT ring around the aorta ring from which it came restored normal PVAT-assisted stress relaxation. The ability of PVAT to assist stress relaxation (a response) is dependent on the PVAT being stretched (a stimulus), whether it is attached or separated from the artery it surrounds.

What these experiments cannot do is discriminate between whether PVAT-assisted stress relaxation is dependent on a relaxant being produced to assist stress relaxation or whether the assistance of stress relaxation by PVAT is solely mechanical in nature. The present study was not undertaken to answer this question, but rather helped us arrive at the next important study of discriminating between these two possibilities. We are committed to determining the mechanism, especially in regards to understanding if vasorelaxant secretions, a hallmark product of PVAT, are necessary. It could be that it is the mechanical properties of PVAT alone – operating in complete independence from the arterial ring – that enables PVAT assisted stress relaxation. In the mouse, the inability to develop aortic PVAT resulted in increased arterial stiffness^24^, while removal of PVAT from the thoracic aorta of Landrace pigs results in ischemic necrosis and dissection^40^. These findings underscore the importance of PVAT as a tissue layer essential to vascular health.

Experiments supported that the structure of PVAT, not its sheer presence, was necessary for its ability to assist stress relaxation. When PVAT mass remained in the tissue bath while attached to the tissue but no longer encompassed the aorta ring, it lost its ability to assist arterial stress relaxation. By contrast, the ability of PVAT to be anticontractile in the presence of a PE challenge was not diminished when PVAT was present but not surrounding the aortic ring. This suggests that stretch in the presence of an active challenge like PE is not required to produce an anticontractile effect; the physical presence of the tissue is sufficient to enable this anticontractile effect. Given that this effect has largely been attributed to either constitutive or agonist-stimulated release of relaxants, this is a logical consideration. Again, the present studies were not designed with the purpose of identifying such substances.

We used a protocol in Fig. 4 different than that in all other experiments, and the outcomes of this protocol are somewhat at odds with the other primary protocol used in our experiments. Two differences exist between the protocols. First, the range of passive tensions applied to the rings was different in the two protocols (up to 6 vs 16 grams). Second, all tissues were challenged with an active stimulus (PE) in between passive tension applications *except* for those studied in Fig. 4 (higher range of passive tensions applied). Because there was no smooth muscle in the PVAT ring alone, this was not a part of the protocol. In the protocol not using a PE challenge, the assistance of aortic ring relaxation by PVAT was modest and not significant when compared to all other experiments (Fig. 4). We do not have a good explanation for this. Inserting a PE challenge into the experiment using the greater range of passive tensions is impractical, as this would require the tissue to remain functional for over 12 hours, longer than a typical isolated bath experiment. If it is this challenge that makes the difference between the outcome of the two stretch protocols, then an active receptor stimulated process is part of the stress relaxation. There are multiple cell types within it that could harbor the α_1_ adrenergic receptor activated by PE: adipocytes, immune cells, fibroblasts, adipogenic precursors, to name a few.

We recognize some limitations of the present study. First, we used a tension range on PVAT that is the same as that for the isolated aorta, assuming that the PVAT experiences similar tensions to the aorta physiologically. This, however, is something we do not know for sure. Second, the variance of responses (PE contraction especially) were higher for samples with PVAT because the variation in PVAT burden from animal to animal and also from segment to segment was high. We have a way to normalize for the response to PE but we do not yet have ways to normalize for PVAT. Third, we do not know if it is the PVAT mass *vs* placement of that mass (or both) that assists stress relaxation. We attempted removing 50% of the PVAT to determine if this would reduce PVAT-assisted stress relaxation by 50%. However, there are several problems with this approach. Removing 1.5 of the 3 strips of PVAT (so 50%) around the artery is unfair because the 2 lateral and 1 ventral side are not identical in their origin^41^. Removing ½ of each of the three strips of PVAT around the thoracic aorta then removes the PVAT from the vessel. Finally, cutting down the PVAT strip to 50%, moving from the outermost boundary of the PVAT strip inwards toward the adventitia, is difficult. This does not take into account that there is a thin layer of white fat that appears to ‘hold’ these three distinct strips of PVAT together; we are unsure how to remove that to a level of 50%. We were not able to do any of these protocols reproducibly and have the remaining confounding factor that the PVAT would still be placed around the aorta.

Fourth, we recognize that the quantitative ability of PVAT in the thoracic aorta of the Dahl S was not as great as in the Sprague Dawley rat. We used the Dahl S model fed a normal diet and on normal salt to be able to address three issues: (1) to demonstrate that the qualitative effect of PVAT assisting stress relaxation was not strain-dependent (SD vs Dahl S); (2) to address that the qualitative effect of PVAT was not sex dependent (M vs F); and (3) to establish baseline data for future experiments in which a high fat (HF) diet is imposed on the Dahl S model to create a hypertension and increased aortic pulse pressure. With this model, we can study whether hypertension associated with an elevated burden of fat, including PVAT, changes PVAT-assisted stress relaxation. The Dahl S rat is one of the few, if not only, rat strain that reproducibly develops a hypertension when on HF diet^42^.

How PVAT stress relaxes itself is our next step of investigation. Stress relaxation is governed by two mechanisms: viscoelasticity and relaxation of smooth muscle cells^43,44^. In highly smooth muscular/low collagen organs with great compliance, such as the stomach fundus and urinary bladder, both mechanisms are present and result in large stress relaxation, consistent with the tissue’s functional need to keep luminal pressure low a majority of the time. This contrasts with the collagen-rich tissues (*e.g*. tendon) in which the stress relaxation is mostly due to the viscoelasticity of the tissue (porosity & fibers reorganization) resulting in less pronounced time dependence of the tension. A change in tissue composition, such as the addition of PVAT to the artery, would necessarily change the overall viscoelastic properties of the tissue. The present studies clearly place PVAT as a tissue that can mechanosense and mechanorespond. A future goal is to determine if *cellular* function (including vasorelaxant production) is necessary for a ring of PVAT to stress relax.

This collective work introduces a new function of PVAT, that of assisting vascular stress relaxation. This work advances human health because our findings are relevant to multiple physiologies. Every major physiological system depends on the circulation for its function. Moreover, these studies raise the idea that PVAT has more impact than previously thought over vascular function. As such, PVAT’s participation in (patho)physiology needs to be reconsidered.

## Methods

### Animals

The MSU Institutional Animal Care and Use Committee (https://animalcare.msu.edu/iacuc/) approved all protocols used in this study. Protocols were performed in accordance with the standards in “The Guide for the Care and Use of Laboratory Animals” (8^th^ Edition, Revised 2011). MSU is an AAALAC accredited institution (A3955-01). Sprague Dawley male or Dahl S male and female rats were purchased (Charles River Laboratories Portage, MI, USA). All rats were housed in a temperature–controlled room (22 °C) with 12-hour light/dark cycles. Sprague Dawley rats were on Teklad 22/5 Rodent diet (Madison WI, USA), and Dahl S rats on Research Diet D124501 (New Brunswick NJ, USA). All rats received distilled water *ad libitum*. The rats used were randomized to studies. Each N value represents data that came from one (1) animal.

### Isolated tissue bath measurement of isometric contraction

Before tissue removal, rats were given pentobarbital as a deep anesthetic (80 mg kg^−1^, ip). A bilateral pneumothorax was created prior to vessel dissection. The thoracic aorta was dissected from the aortic arch to the diaphragm. The superior mesenteric artery was dissected from the aorta. Tissue dissection took place under a stereomicroscope and in a Silastic®-coated dish filled with physiological salt solution (PSS) containing [mM: NaCl 130; KCl 4.7; KH_2_PO_4_ 1.18; MgSO_4_ • 7H_2_O 1.17; NaHCO_3_ 14.8; dextrose 5.5; CaNa_2_EDTA 0.03, CaCl_2_ 1.6 (pH 7.2)]. The endothelium was left intact. For creating rings of aorta (~5 mm length) or superior mesenteric artery (~3–5 mm length) with and without PVAT, the vessel was guided onto a wire that was embedded into the dish silastic to allow for tissue revolution such that the vessel could be completely cleaned of PVAT. In some experiments, all PVAT was dissected from all but the ends of the ring such that the mass of PVAT was still present but did not encompass the arterial ring. Samples were termed aortic ring +PVAT (+PVAT), aortic ring −PVAT (−PVAT) or aortic ring +/−PVAT (+/−PVAT).

For creating separated rings of the vessel and its surrounding PVAT, a section of the vessel +PVAT was stood on its end in the silastic dish and two small insect pins inserted into the lumen of the aorta to hold the vessel open. While gently holding the PVAT layer away from the adventitia, small vannas scissors were used to sever connections around the circumference of the vessel between the PVAT and aorta. The whole ring was then flipped vertically and this process repeated. The separated ring of PVAT (PVAT ring) was then lifted off the aorta. In some experiments, the PVAT ring was placed back around aortic ring in its original position. This was termed aortic ring +PVAT ring removed and added back.

All tissues were mounted onto two L-shaped stainless-steel hooks. Rings were mounted in warmed (37 °C) and aerated (95% O_2_, 5% CO_2_) tissue baths (30 mL PSS) on Grass isometric transducers (FT03; Grass instruments, Quincy, MA, USA) connected to a 4 channel PowerLab (ADInstruments, Colorado Springs, CO, USA). Two independent investigators performed these experiments on tissue bath set ups in two different rooms. Sample types (aortic ring +PVAT, aortic ring −PVAT, aortic ring +/−PVAT, PVAT ring- or aortic ring +PVAT ring removed and added back) were randomized daily into one of four different baths. Care was taken to apply no tension on the tissue prior to initiation of the experiment.

#### Protocol I: Stress relaxation with PE challenge: arterial ring +, − and +/−PVAT

All rings started at a tension of 0 grams. Aortic rings were challenged with a cumulative passive tension application of 0.25, 0.5 (0.25 g added), 1 (0.5 gram added), 2 (1.0 gram added), 4 and 6 grams (2 grams each added). Superior mesenteric artery rings were subjected to a smaller range of tensions from 0.1, 0.2 (0.1 g added), 0.4 (0.2 gram added), 0.8 (0.4 gram added), 1.6 (0.8 gram added) and 2.4 g (0.8 gram added). Tissues were allowed to relax to this stretch for 30 minutes; the tension achieved at this time was recorded. Tissues were then challenged with a maximum concentration of PE (10^−5^ M), the response allowed to plateau and response recorded. Tissues were washed identically and repeatedly through 30 minutes to achieve a stable baseline. At this time, the next tension step was placed. At the end of the experiment, tissues were weighed and some processed for histology (described below).

In some experiments, PVAT was fully separated from the aorta, and then the two separate rings of tissue placed back together as described above. This manipulation allowed testing whether direct physical connections were necessary for the endpoints measured. At the end of the experiment, tissues were weighed and some processed for histology (described below).

#### Protocol II: Stress relaxation with no PE challenge

Rings started at a tension of 0 grams. They were challenged with a cumulative passive tension application of 0.5, 1.0 (0.5 g added), 2 (1.0 gram added), 4, 6, 8, 10, 12, 14 and 16 grams (2 grams each added step). Tissues relaxed to this stretch for 25 minutes; the tension achieved at this time was recorded. Tissues were washed 2x over 5 minutes. At this time, the next tension step was placed.

### Histology

In some experiments, rings were thoroughly washed and removed from the hooks on which they were placed in the tissue bath. Tissues were fixed in 10% formalin overnight, switched to 30% ethanol and taken to Investigative Histopathology Services (https://humanpathology.natsci.msu.edu/) on the campus of MSU for section cutting to provide slides with 8–10 micron thick sections. Slides were processed with a modified Verhoeff Van Gieson stain and were blinded to the processor of the slides.

### Materials

Phenylephrine hydrochloride was purchased from Sigma Chemical Co (St. Louis, MO, USA). Formalin (10%) buffered phosphate solution was purchased from Fischer Scientific (Waltham, MA USA).

### Data analyses and statistics

For each endpoint, the N value represents the number of individual animals used and this is replicated by showing the number of individual symbols in the scatter plots. Data, as line graphs or in bar graphs with scattered points, are reported as means ± SEM. The magnitude of relaxation after each stretch step was recorded 25–30 after minutes after the initial stretch. Overall stress relaxation was quantified in two ways. First, the area under the curve (from 0 to the maximum amount of passive tension pulled) was measured. This value was determined for each individual response, averaged, and then compared as a whole between experimental groups [arterial (aortic or superior mesenteric) ring +PVAT, arterial ring −PVAT, aortic ring +/−PVAT, PVAT ring or aorta ring +PVAT ring removed and added back]. Second, the maximum passive tensions achieved were compared. For measuring the response to PE that followed a stretch step, the contraction elicited was allowed to plateau and this response considered the maximum. When two groups are compared, an unpaired Students *t* test was used as while the samples came from the same rat, they were different tissues. All variances passed variance equality tests prior to Student *t* test. When three groups were compared, a one-way ANOVA followed by Tukey’s posthoc was used as long as variances were homogeneous as determined by the Brown Forsythe test. A p value of <0.05 was considered statistically significant and significant p values are reported in the figures.

## Data Availability

Original data produced will be shared upon request. There are no database worthy data produced within this manuscript.
